# Performance of resting metabolic rate estimation equations in obese patients

**DOI:** 10.1186/1758-5996-7-S1-A231

**Published:** 2015-11-11

**Authors:** Milene Moehlecke, Manoel Roberto Maciel Trindade, Ana Carolina Mazzuca, Carina Andriatta Blume, Jakeline Rheinheimer, Cristiane Bauermann Leitão

**Affiliations:** 1 UFRGS, Porto Alegre, Brazil

## Background

Weight gain may be associated with an imbalance between energy intake and energy expenditure. The resting metabolic rate (RMR) is the main component of total energy expenditure, and is related mainly to lean mass (LM), as well as to other factors such as fat mass (FM), age, sex and genetic factors. A RMR lower than expected may be a risk factor for weight gain. RMR is estimated by equations that use patient's weight, sex, age and height to calculate energy needs. Several studies have shown that these equations have a poor agreement with RMR measured by indirect calorimetry (IC) in obese patients once their excess fat-free mass (FFM) is usually not taken into account.

## Objective

To evaluate the accuracy of five equations in predicting RMR in obese subjects. Results were compared with measured RMR (mRMR) determined by IC.

## Materials and methods

**Figure 1:**
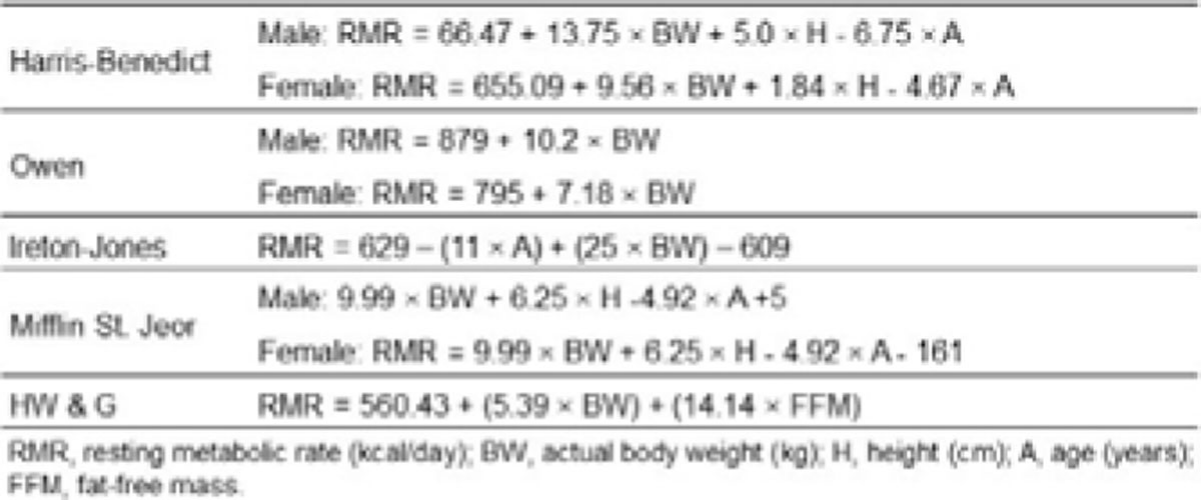
Prediction equations for comparison with indirect calometry in obese subjects.

Cross-sectional study was conducted in obese Southern Brazilian volunteers recruited from community. Body mass index (BMI) was calculated by dividing weight (in kilograms) by squared height (in meters). Body composition was evaluated by dual-energy X-ray. RMR was measured by IC (Weir equation) and estimated (eRMR) by Mifflin–St. Jeor, Owen, Harris-Benedict, Ireton-Jones and Horie-Waitzberg & Gonzalez (H & WG) equations (Figure 1). The latter takes into consideration the FFM. Equations performance were determined by bias (mean difference between mRMR and eRMR); precision (standard deviation of bias) and by accuracy (percentage of estimates within 5% of mRMR).

## Results

**Figure 2:**
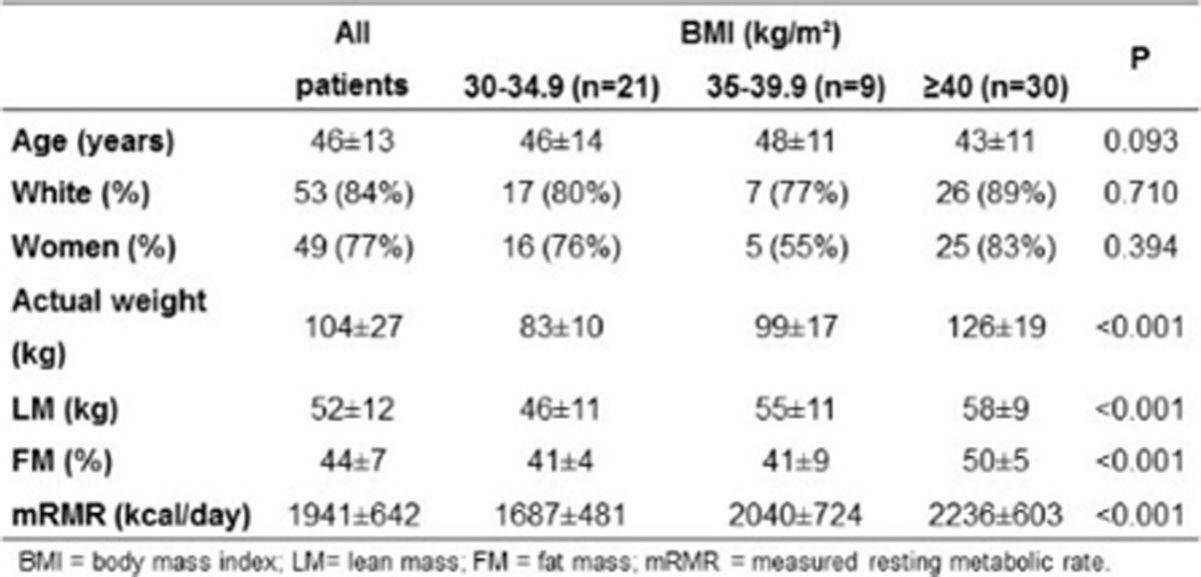
Obese patients distributed by anthropometric and body composition parameters.

**Figure 3:**
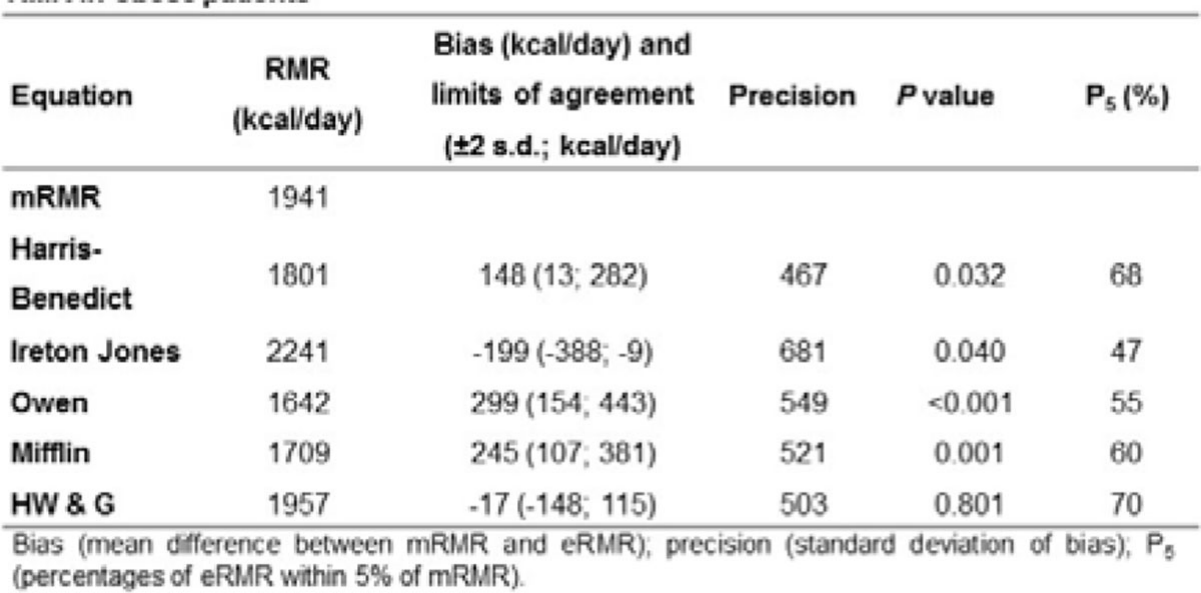
Comparison between estimated RMR from several equations and measured RMR in obese patients.

**Figure 4:**
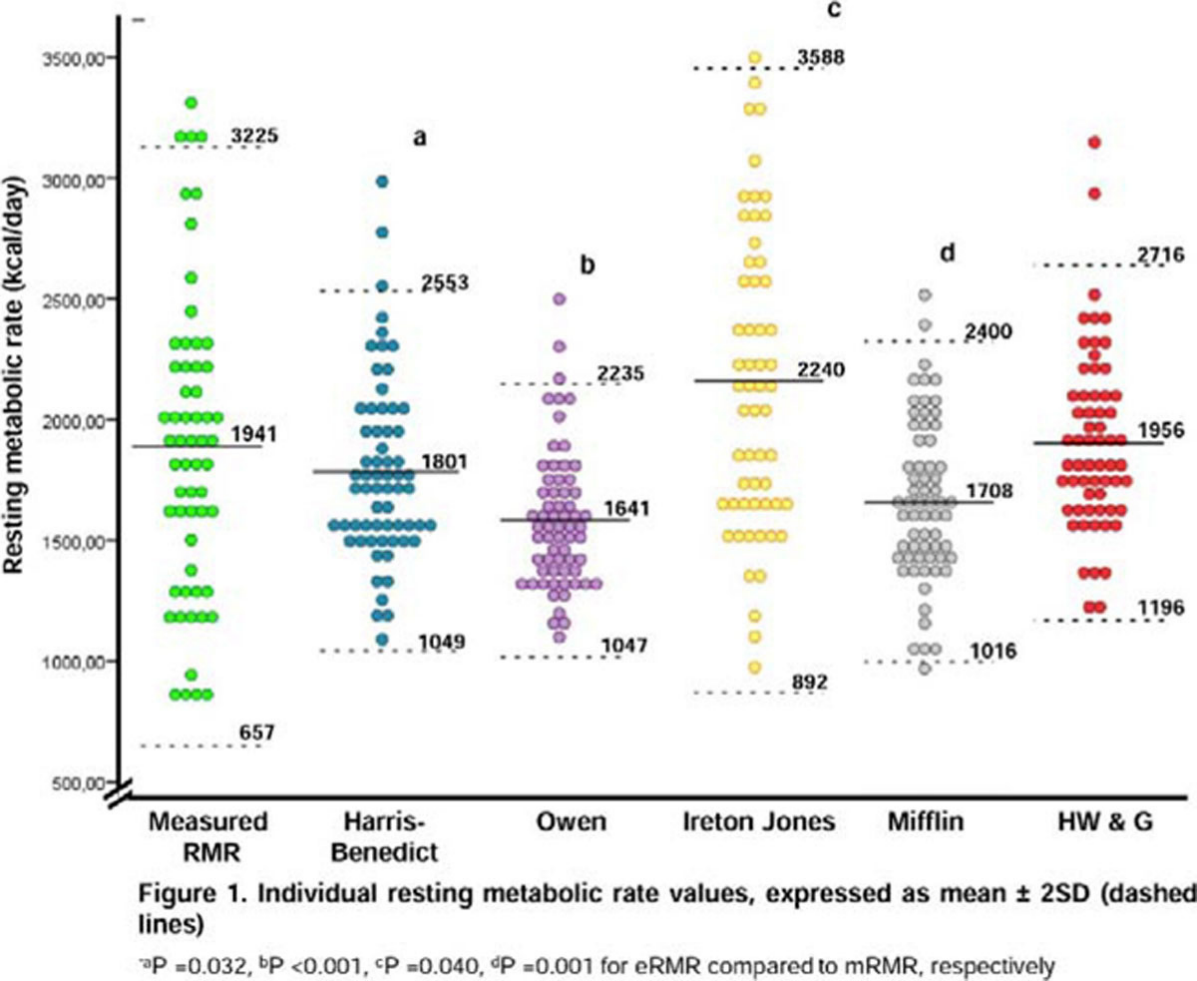
Individual resting metabolic rate values, expressed as mean ± 2SD (dashed lines)

Sixty individuals (46 women [75%], 48 white [84%]) aged 46±13 yrs. (range, 21-83 yrs.) were evaluated. Overall, mRMR was 1941±642 kcal/day. mRMR increased along with BMI (Figure 2), but the association was lost when corrected for LM (P=0,859). H & WG equation was the only equation unbiased (P=0.801) (Figure 3). The Harris-Benedict, Owen and Mifflin–St. Jeor equations were biased overall toward underestimation, while Ireton-Jones equation was biased toward overestimation (Figure 4). Bias was significantly higher in women for Harris-Benedict, Mifflin St. Jeor and Owen equations. Accuracy to estimate RMR at ±5% was suboptimal for all equations, except for H & WG.

## Conclusion

In this sample of obese subjects, the available RMR estimate equations that do not take into account the FFM have poor accuracy when compared with mRMR.

